# A Tubular Flexible Triboelectric Nanogenerator with a Superhydrophobic Surface for Human Motion Detecting

**DOI:** 10.3390/s21113634

**Published:** 2021-05-23

**Authors:** Jianwei Wang, Zhizhen Zhao, Xiangwen Zeng, Xiyu Liu, Youfan Hu

**Affiliations:** 1 Hunan Institute of Advanced Sensing and Information Technology, Xiangtan University, Xiangtan 411105, China; 201821521363@smail.xtu.edu.cn; 2 Key Laboratory for the Physics and Chemistry of Nanodevices, Center for Carbon-Based Electronics, Department of Electronics, Peking University, Beijing 100871, China; 1701111240@pku.edu.cn (Z.Z.); zengxiangwen@pku.edu.cn (X.Z.); liuxiyu@pku.edu.cn (X.L.)

**Keywords:** triboelectric nanogenerator, wearable electronics, self-powered sensing, stretchable, superhydrophobic surfaces

## Abstract

The triboelectric nanogenerator (TENG) is a newly arisen technology for mechanical energy harvesting from the environment, such as raindrops, wind, tides, and so on. It has attracted widespread attention in flexible electronics to serve as self-powered sensors and energy-harvesting devices because of its flexibility, durability, adaptability, and multi-functionalities. In this work, we fabricated a tubular flexible triboelectric nanogenerator (TF-TENG) with energy harvesting and human motion monitoring capabilities by employing polydimethylsiloxane (PDMS) as construction material, and fluorinated ethylene propylene (FEP) films coated with Cu as the triboelectric layer and electrode, serving in a free-standing mode. The tube structure has excellent stretchability that can be stretched up to 400%. Modifying the FEP films to obtain a superhydrophobic surface, the output performance of TF-TENG was increased by at least 100% compared to an untreated one. Finally, as the output of TF-TENG is sensitive to swing angle and frequency, demonstration of real-time monitoring of human motion state was realized when a TF-TENG was worn on the wrist.

## 1. Introduction

Recently, with the rapid development of a new generation of digital information technology, the implementation of various portable and wireless electronic devices has changed the way people live [1,2,3,4], and one consequence is that the energy supply of these electronic devices has become a critical challenge. As a new energy harvesting technology, triboelectric nanogenerators (TENGs) are based on the coupling effect of triboelectrification effect and electrostatic induction [5], which was first invented by Wang and coworkers in 2012 [6] and was widely used to convert randomly distributed, irregular, weak, and low-frequency mechanical energy into electric power [7,8,9,10,11,12]. The TENGs have four fundamental working modes, namely contact-separation mode, sliding mode, single-electrode mode, and free-standing mode [13,14,15,16]. With proper working modes, TENGs can fit into different application scenarios, to serve as, for example, micro/nano energy harvesters [17,18,19] and self-power sensor systems [20,21,22,23].

The generation of triboelectric charges during the triboelectrification process is highly dependent on the two contact materials’ relative ability to gain or lose electrons, or their relative positions in the triboelectric series [24]. Recently, many efforts have been made to develop or modify materials to promote the performance of TENGs [25,26,27]. Most TENGs are based on a solid–solid contact, causing physical wear of the device with surface damages, which would lead to output performance degradation with time. Instead, liquid–solid interface-based TENGs can provide higher durability, flexibility, and a larger effective contact area for a higher output performance [28,29,30]. Lately, many wearable TENGs have been reported [31,32,33,34,35,36] for energy harvesting from the body or self-powered sensing for body movements; however, there is still lots of room for improving their wearing comfortability, lowing manufacturing cost, and extending application scenarios.

In this work, we fabricated tubular flexible TENGs (TF-TENGs) with excellent stretchability and super hydrophobicity that were introduced at liquid–solid interfaces for triboelectrification in a free-standing mode. The effects of swing angles and frequencies on the output performance of TF-TENGs were first checked by using Cu pellets as the other triboelectric layer rolling in the tube. Then, after the treatment of super hydrophobicity, pure water could roll easily in the TF-TENGs, demonstrating a 100% performance improvement compared to the untreated one. Finally, a TF-TENG was wrapped on the wrist for human motion state detection, revealing its application potentials in the field of smart sports.

## 2. Materials and Methods

**Figure 1:**
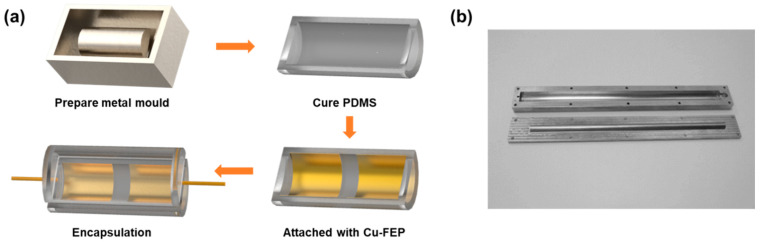
(**a**) Schematic diagram of the fabrication process. (**b**) Custom-made metal molds.

Figure 1a shows a schematic diagram of the fabrication process of a TF-TENG, which consists of a (polydimethylsiloxane) PDMS tube (with a wall thickness of 3 mm) as the encapsulation layer and fluorinated ethylene propylene (FEP) films coated with Cu attached on the inner surface to serve as one of the triboelectric layers and the electrode, respectively. First, metal molds were prepared, with their designs shown in Figure 1b. The upper metal mold has a length of 26 cm and a width of 3 cm. The inner groove has a length of 24 cm and a hollow semi-cylinder with a diameter of 1.4 cm. The lower metal mold has a length of 26 cm and a width of 3 cm. The protrusion is a solid half-cylinder with a length of 22 cm and a radius of 0.8 cm. Then, PDMS base agent (vinyl silicone oil monomer, polysilicon dioxide methyl silicone oil) and curing agent (vulcanization agent, silicon dioxide, platinum catalyst) were mixed at a weight ratio of 1:1.2. The mixtures were stirred by a centrifugal mixer (AR-100, Thinky) under a rotation speed of 1500 rpm for 15 min to ensure that the mixtures were well blended and degassed. After pouring the precursor of PDMS in the metal mold and curing it at 100 °C for 3 h, PDMS film with a half-tube structure was obtained. It should be mentioned that the metal mold surface was treated with trimethylchlorosilane in advance to provide a non-stick surface during PDMS film molding. Then, fluorinated ethylene propylene (FEP) films coated with Cu (Cu-FEP films) were attached to the inner surface of the PDMS tube, in which the FEP film was chosen as the triboelectric layer because of its low cost, light weight, and suitability as almost the best electronegative material [37]. The Cu-FEP films were prepared by: (1) using DC sputtering (PVD75, Kurt J. Lesker) with a power of 1 kW to deposit 200 nm thick Cu on an FEP film surface; (2) cutting into a size of 10 cm × 1.2 cm by using a laser cutting machine (PLS6MW, Universal Laser Systems); (3) cleaning with deionized water and blowing dry with nitrogen. Finally, another half tube of PDMS film was used to cover and encapsulate the whole structure with two electrical wires stuck out at the two ends to make electrical connections between the Cu electrodes at the backside of the FEP film and the measurement equipment.

For superhydrophobic surface treatment of the Cu-FEP film, the Cu-FEP film was first cleaned using deionized water and blow-dried with nitrogen gas. The cleaned surface was then sprayed with a layer of base coat of NeverWet^TM^ multi-surface liquid repelling treatment (Rust-Oleum, USA) containing methyl isobutyl ketone, butyl acetate, and mineral spirits. The base coat was sprayed uniformly 2–3 times over the surface at a distance of around 15 cm, for about 3–4 s each time. Then, the Cu-FEP film was left to dry at normal room temperature for 30 min. After that, a layer of topcoat containing silica nanoparticles suspended in acetone was sprayed onto the surface. The coated Cu-FEP film surface was left to dry for 30 min to achieve a superhydrophobic surface. The surface was kept for further drying in normal laboratory conditions for 12 h before using it.

For characterization, a Carl Zeiss Axio Imager optical microscope was used to record the surface morphology of the Cu-FEP film. The triboelectric output signals were measured by a programmable electrometer (Keithley 6514) and a data acquisition device (NI PXI-6259).

## 3. Results and Discussion

### 3.1. As-Fabricated TF-TENG and Its Strechability

**Figure 2:**
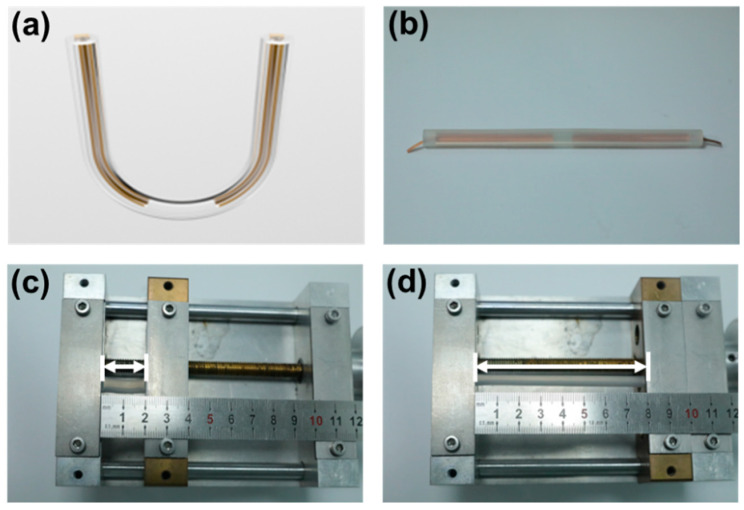
(**a**) The schematic illustration and (**b**) a photograph of an as-fabricated TF-TENG. (**c**) A PDMS tube at original length, and (**d**) when it was stretched to 400%.

Figure 2a sketches the structure diagram of a TF-TENG, and Figure 2b is the photograph of an as-fabricated TF-TENG with a length of 24 cm and a diameter of 14 mm. It shows that the PDMS tube is semitransparent and two Cu-FEP films are attached separately on its inner surface. We first tested the stretchability of the structure, which was measured by using a homemade sample stage as illustrated in Figure 2c,d. Due to the limitation of movable range of the sample stage, we cut the PDMS tube and clamped two ends of the tube into the sample stage with a suspended length of 2 cm. It can be stretched up to 8 cm, 400% of the original length, without mechanical fracture, which demonstrates its excellent stretchability.

### 3.2. Working Principle of TF-TENG

**Figure 3:**
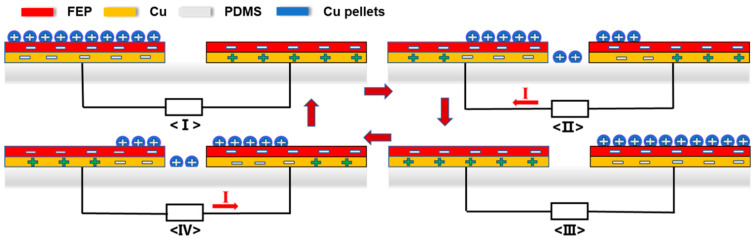
The schematic illustration of the working principle of the TF-TENG.

The working principle of the TF-TENG is depicted in Figure 3, which is based on the freestanding mode. First, as an example to simplify the case, we used Cu pellets to work as the other triboelectric layer rolling on the FEP films. After the first cycle of rolling, as shown in Figure 3<I>, due to the triboelectrification effect, positive charges are generated on the surfaces of the Cu pellets and the quantity of these positive charges is equal to the sum of the negative charges generated on two FEP films. At the same time, charges that are induced by electrostatic induction in the left and right Cu electrodes are equal in number while opposite in sign. Then, when the positively charged Cu pellets roll to the right FEP film by external force, electrons flow from the left electrode to the right electrode, generating current from the right to the left through the external load (Figure 3<II>). Until all the Cu pellets move to the right side, the transferred charges between the two electrodes will reach the maximum, as shown in Figure 3<III>. Then, a backward moving of these Cu pellets from right to left results in a reverse current in the external load (Figure 3<IV>). When the Cu pellets reach the original position on the left FEP film, all of the negative charges in the right electrode will be driven to the left electrode (Figure 3<I>). By cycling the moving process of the Cu pellets between the surfaces of two FEP films, an alternating current is generated.

### 3.3. Output Performance Characterization of the TF-TENG

**Figure 4:**
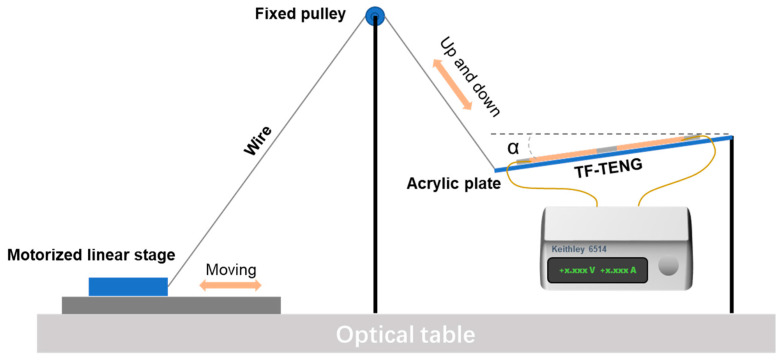
The schematic illustration of the experimental setup for output performance characterization of the TF-TENG.

In the as-fabricated TF-TENG, two Cu-FEP films with a size of 10 cm × 1.2 cm were attached to the PDMS tube inner surface with a separation distance of 2 cm, and Cu pellets with a diameter of 2 mm were first enclosed into the PDMS tube as the freestanding triboelectric layer to characterize the output performance of the TF-TENG. Figure 4 shows the schematic illustration of the experimental setup for performance characterization. To introduce a swing motion into the TF-TENG, the TF-TENG was first placed on an acrylic plate. One end of the plate was fixed, and the other end could swing up and down, which was controlled by a motorized linear stage (MTN300CC, Newport) that was connected via a setup of a wire and a fixed pulley. The swing angle α of the device is defined as the angle between the orientation of the acrylic plate and the horizontal line, which is indicated in Figure 4.

**Figure 5:**
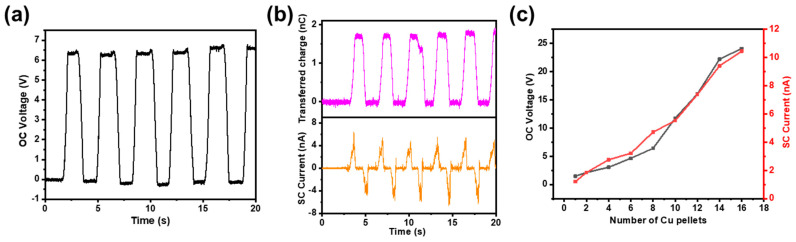
(**a**)The OC voltage, (**b**) transferred charges and SC current of a TF-TENG. (**c**) The relationship of the OC voltage, SC current and the number of Cu pellets.

The output characteristics of open-circuit (OC) voltage, short-circuit (SC) current, and transferred charges of the TF-TENG were measured at a swing angle of 5° and a swing frequency of 0.25 Hz. As shown in Figure 5a, the OC voltage of the FT-TENG is about 6.5 V. The quantity of transferred charges and SC current illustrated in Figure 5b are 1.8 nC and 5 nA, respectively. Figure 5c shows the relationship between the number of Cu pellets and the electric output performance of the TF-TENG. When the number of Cu pellets increases from 1 to 16, the OC voltage increases from 1.5 V to 24.1 V, and the SC current also increases from 1.2 nA to 10.5 nA, which shows a certain degree of linear relationship. This is because when the number of Cu pellets increases, the effective contact areas between the freestanding layer and the surface of the FEP film increase, which increases the quantity of transferred charge correspondingly with an approximately linear relationship.

**Figure 6:**
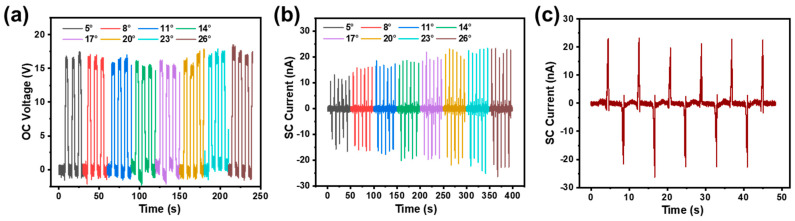
(**a**) OC voltage and (**b**) SC current of the TF-TENG under different swing angles. (**c**) Enlarged SC current curve under the swing angle of 26°.

In addition, the influence of different swing angles on the electric output of the TF-TENG was investigated. When the swing angle increases from 5° to 26°, the OC voltage of the device is almost the same, but the SC current increases significantly, as shown in Figure 6a,b. In this case, since the quantity of transferred charges is the same when the same number of Cu pellets are used, the OC voltage does not change as it is determined by the surface charge density and the electrode separation distance. The rolling speed of the Cu pellet is positively correlated with the swing angle, which leads to more electric charges passing through the external load per second when the swing angle increases, resulting in a larger SC current. The SC current under a swing angle of 26° is enlarged in Figure 6c to reveal its good reproducibility.

**Figure 7:**
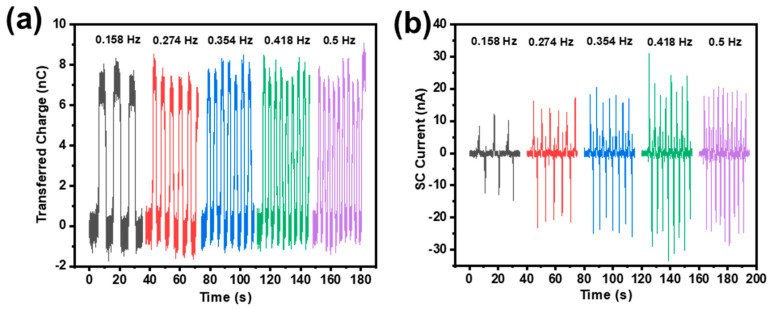
(**a**) Transferred charges and (**b**) SC current under different swing frequencies.

The relationship between the working frequency and the output performance has also been studied, as exhibited in Figure 7. Under the condition that the other influencing factors remain unchanged (swing angle, number of Cu pellets, electrode separation distance, etc.), when the swing frequency increases from 0.158 Hz to 0.5 Hz, it can be seen that the quantity of transferred charges is about 8 nC and stays the same, while the SC current increases from 10 nA to 20 nA. The quantity of charge transferred remains unchanged because the effective contact area between the triboelectric layer and the FEP film has not changed. Therefore, an increase in frequency will only lead to an increase in the speed of charge transfer and thus an increased SC current.

**Figure 8:**
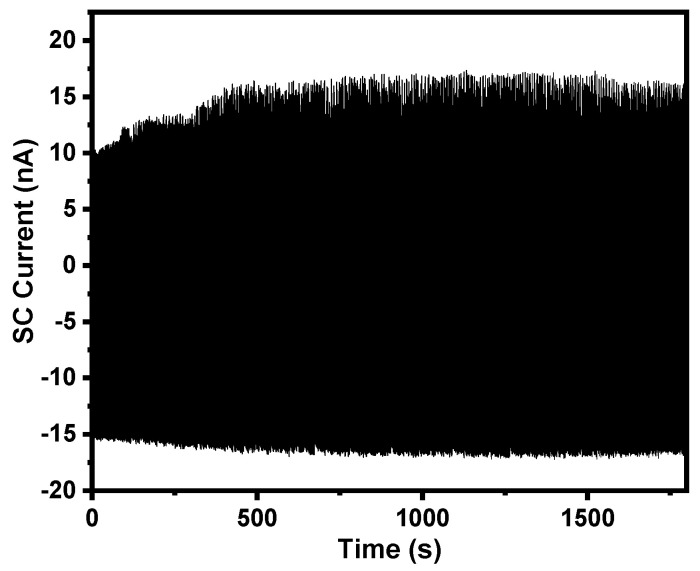
SC current of a TF-TENG under 3500 cycle tests at a swing angle of 15° and a swing frequency of 2 Hz.

In order to evaluate the stability of the device, we measured the SC current of the TF-TENG for 3500 continuous working cycles with a swing angle of 15° and a swing frequency of 2 Hz, as shown in Figure 8. The output current almost maintains at a constant value, indicating that the TF-TENG has good stability and durability.

### 3.4. Superhydrophobic Surface Treatment of the TF-TENG

**Figure 9:**
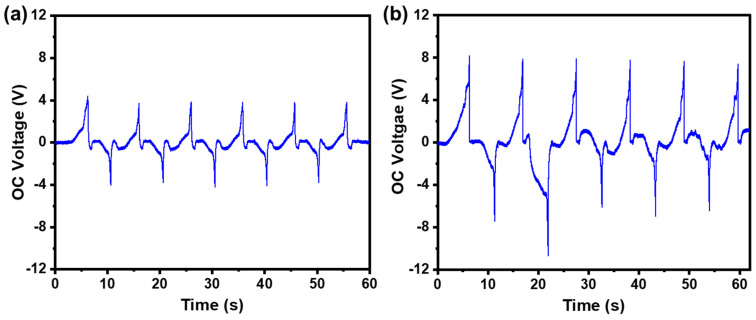
The OC voltage of a TF-TENG (**a**) before and (**b**) after superhydrophobic surface treatment.

The Cu pellets with a certain weight cannot roll easily in a curved tube when the TF-TENG is worn on the human body. Hence, pure water was injected into the PDMS tube to replace Cu pellets and serve as the triboelectric layer material. Pure water has good shape adaptability and can flow under a small deflection angle, which can perfectly solve the problem met by Cu pellets to realize lightweight and more sensitive TF-TENGs. As shown in Figure 9a, when 2 mL pure water was injected into the PDMS tube, an OC voltage of 4 V was obtained. To further elevate the responsibility of the device under a small mechanical disturbance, the surface of the FEP films were treated with NeverWet^TM^ multi-surface liquid repelling treatment (Rust-Oleum, USA) to realize a superhydrophobic surface. After the treatment, the OC voltage of the TF-TENG was increased to 8 V, as shown in Figure 9b, which is double the output of the untreated one.

**Figure 10:**
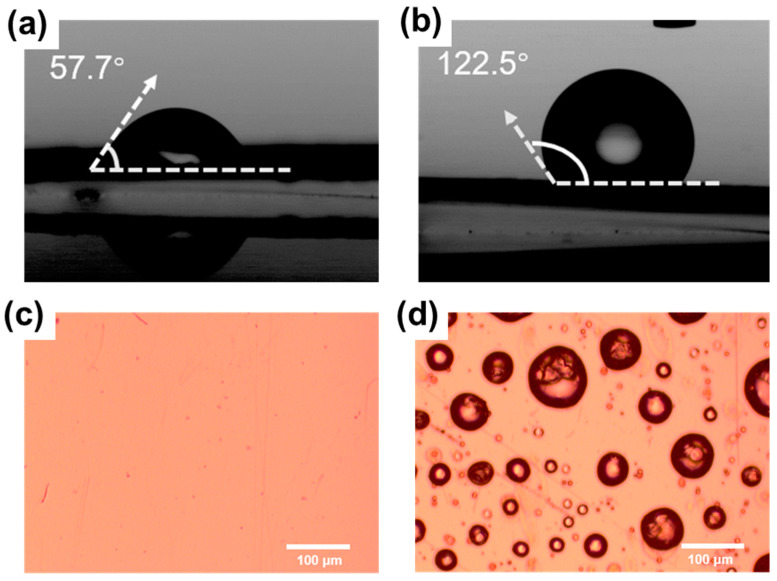
Contact angle measurement results (**a**) before and (**b**) after superhydrophobic surface treatment. Optical image of the FEP film surface (**c**) before and (**d**) after superhydrophobic surface treatment.

To understand the improvement in performance, we checked the surface of the FEP film. First, we measured the contact angle of the FEP film before and after the superhydrophobic treatment, with the results shown in Figure 10a,b. It is clear that the contact angle of the pure water on the FEP film surface is increased from 57.7° to 122.5°, demonstrating a good superhydrophobic effect. To further check the surface morphology with optical microscope, Figure 10c,d show that for an untreated FEP film, the surface is almost clear, while for a treated one, there are randomly distributed microparticles on the surface that were introduced by the sprayed NeverWet^TM^ solution, which introduces the superhydrophobic behavior [38]. These almost uniformly distributed silica microparticles also increase the surface roughness and dielectric constant, which simultaneously further improve the triboelectric charge density on the FEP film surface [39,40,41,42], and thus the output performance of the TF-TENG.

### 3.5. Application of the TF-TENG in Human Motion Monitoring

**Figure 11:**
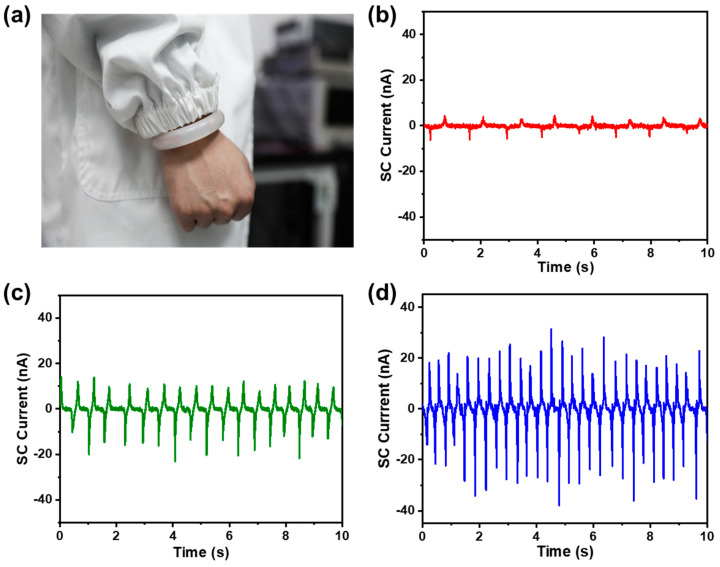
(**a**) Photograph shows a TF-TENG worn on the human wrist. SC current of a TF-TENG when the wearer is swinging the arm at (**b**) 0.8 Hz, (**c**) 1.8 Hz, and (**d**) 2.7 Hz.

Generally, when people move at different speeds, the state of motion, including the frequency and amplitude of arm swings, is different. Accordingly, to prove the capability of the TF-TENG as a sensor for human motion monitoring, we wrapped the device around the wrist, as shown in Figure 11a, and recorded the electrical signal changes when the human body is in different motion states under a natural swing of the arm, as shown in Figure 11b–d. First of all, under different motion states, the motion frequencies are distinguished from each other, and it is also observed that the output SC current increases as the motion frequency increases, which makes the difference more remarkable. When the wearer swung the arm at 0.8 Hz, the measured SC current of the TF-TENG was 4 nA, while when the wearer changed to swing the arm at 1.8 Hz and at 2.7 Hz, the SC current increased to 10 nA and 20 nA, respectively. This means that the TF-TENG is sensitive enough to capture different body motions and can be used for real-time analysis of the human motion state.

**Figure 12:**
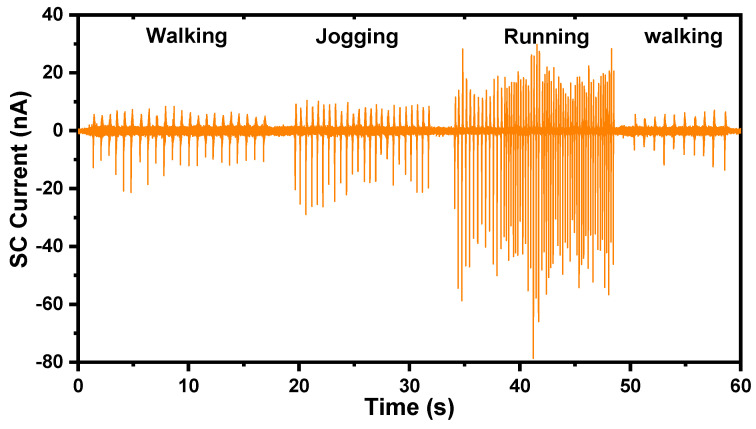
SC current of the TF-TENG when the human body is in different motion states.

Figure 12 shows the recorded signal of SC current when the human body is in different motion states, including walking, jogging, and running. The frequency and amplitude of the sigal both change with different motion states; thus, it is easy to distinguish between them. The demonstration reveals that the TF-TENG can be used as a self-powered sensor for human motion monitoring, and expands the TENG’s application in self-powered sensing systems for smart sports.

## 4. Conclusions

In summary, we designed a tubular flexible TENG with mechanical energy collection and human motion monitoring functions. PDMS was used as the construction material to provide 400% stretchability, and FEP film and pure water constituted the triboelectric layers of the TF-TENG. Through superhydrophobic surface treatment, the output performance of the TF-TENG doubled due to the increased surface charge density, and also the device became easier to be triggered. The TF-TENG is lightweight and can be comfortably worn on the wrist, by which different motion states, such as walking, jogging, and running, can be monitored and distinguished in real time, demonstrating its application potential in the field of smart sports. Currently, the TF-TENGs are wired to the external electrometer for signal recording. A fully integrated wireless system with the capabilities of signal recording, processing, analysis, and wireless data transmission will further elevate the adaptability and conveniences of this technology to fit into diversified application scenarios.

## Data Availability

Not applicable.
